# Host-driven remodelling of the Staphylococcus aureus cell envelope: mechanisms, consequences and therapeutic implications

**DOI:** 10.1099/mic.0.001718

**Published:** 2026-06-08

**Authors:** Elizabeth V. K. Ledger

**Affiliations:** 1 School of Microbiology, University College Cork, Cork, Ireland; 2 APC Microbiome Ireland, University College Cork, Cork, Ireland

**Keywords:** cell membrane, cell surface, cell wall, host environment, *Staphylococcus aureus*

## Abstract

*Staphylococcus aureus* is a leading cause of life-threatening infections worldwide and remains difficult to treat despite the availability of antibiotics. The bacterial cell envelope is central to *S. aureus* pathogenesis as it forms the primary interface with host tissues and the immune system and is the target for many of our most successful antibiotics. Much of our current understanding of staphylococcal cell envelope biology derives from studies performed under standard laboratory conditions that do not accurately reflect the complex environment encountered during infection. Increasing evidence indicates that *S. aureus* profoundly remodels its cell envelope in response to host-derived stresses, generating a surface architecture that differs markedly from that observed *in vitro*. This review discusses how host-associated factors, including nutrient limitation, antimicrobial peptides, oxidative stress, altered pH and hypoxia, drive extensive remodelling of both the bacterial membrane and the cell wall. These changes affect multiple aspects of the cell envelope, including phospholipid and fatty acid composition, peptidoglycan thickness, teichoic acid abundance and modification and capsule production. Importantly, this host-induced cell envelope remodelling has important functional consequences, altering antibiotic tolerance, susceptibility to immune killing and interactions with host cells and tissues. Because these changes are non-genetic and reversible, they are unlikely to be detected by conventional diagnostic approaches; however, they may play an important role in treatment failure and persistent infection.

## Introduction

*Staphylococcus aureus* causes over 1 million deaths every year and is the leading cause of fatal bloodstream infections in over 135 countries [1]. All clinical trials of human vaccines have failed, and so despite extensive efforts, there is currently no effective human vaccine [2]. In the case of staphylococcal bloodstream infections, treatment involves long courses of intravenous antibiotics which must be carefully selected based on the antibiotic susceptibility profile of the strain. However, despite appropriate antibiotic therapy, *S. aureus* bacteraemia is associated with a 20–30% mortality rate and often causes long-lasting complications in survivors, including cardiovascular damage as well as chronic and/or relapsing infections [3]. This high rate of relapse highlights that *S. aureus* is frequently able to withstand both host defences and antibiotic therapy.

The staphylococcal cell envelope is an important target for antibiotics and vaccines as it is essential for bacterial viability and is at the interface of pathogenesis, immunity and antimicrobial susceptibility [4]. As a Gram-positive bacterium, the cell envelope consists of a single phospholipid bilayer, surrounded by a thick (~20 nm) layer of peptidoglycan [5]. It also contains teichoic acids, which are either anchored to the membrane [lipoteichoic acid (LTA)] or the peptidoglycan [wall teichoic acid (WTA)], as well as many proteins, which can either be covalently attached to the peptidoglycan or surface associated [6,8]. Finally, many strains synthesize a polysaccharide capsule, which is also covalently linked to the peptidoglycan [9].

The surface proteins are crucial for pathogenesis, playing varied roles in colonization and infection, and, along with WTA, are the main staphylococcal targets recognized by human antibodies, leading to phagocytosis and killing by immune cells, particularly neutrophils [10,11]. Peptidoglycan synthesis is the target for many antibiotics, including the commonly used beta-lactams and the last resort antibiotic vancomycin, while the membrane and membrane-bound lipid precursors of peptidoglycan are the target of daptomycin [12,14]. Therefore, understanding the cell envelope structure and properties is essential to understanding how *S. aureus* interacts with the host, as well as how the bacterium survives exposure to antibiotics and the immune system. However, much of our current understanding of staphylococcal cell envelope biology comes from experiments performed under laboratory conditions, which do not replicate the environment encountered during infection.

There is a growing appreciation that the host environment significantly affects the structure of the cell envelope and that this has profound consequences for *S. aureus* pathogenesis, immune evasion and antibiotic susceptibility. Therefore, this review focuses on how the host environment induces non-genetic changes in the cell surface which are not seen under standard laboratory conditions and the implications of this for host–pathogen interactions and the way we treat staphylococcal infections.

## Host factors that affect staphylococcal physiology

One important host factor that affects bacterial surface physiology is the nutrient availability within the host environment. During an infection, bacteria must obtain all the nutrients they need to grow from the host environment. Therefore, to restrict pathogen proliferation, the host sequesters important nutrients in a concept known as nutritional immunity [15]. *S. aureus* needs transition metals such as iron, zinc and manganese for a range of processes, including carbohydrate and amino acid metabolism and protection against oxidative stress [16,17]. The concentrations of these ions in the host are tightly regulated [15,18,20]. For example, the majority of iron in the host is stored intracellularly or tightly bound to host iron-binding proteins, including haemoglobin, transferrin and ferritin [21], while calprotectin, an abundant protein in neutrophils, chelates additional metal ions including zinc and manganese [16]. In addition, carbon and amino acid availability also varies markedly between infection sites and can become limiting as bacterial densities increase, causing both slow growth and gene expression in host tissues/fluids to be very different from that observed in media [22,23].

The immune system also imposes stresses which affect the cell envelope. Infection sites are rich in antimicrobial peptides (AMPs) which are produced by multiple host cell types and are a key defence against *S. aureus*. These include cationic AMPs such as cathelicidins (e.g. LL-37) and defensins, which are secreted by neutrophils, epithelial cells and other immune cells [13]. Many AMPs exert their antibacterial activity through electrostatic interactions with the bacterial surface, leading to membrane disruption. Neutrophils also produce high concentrations of reactive oxygen species (ROS) via nicotinamide adenine dinucleotide phosphate oxidase, which produces superoxide from molecular oxygen [24]. This superoxide dismutates to form hydrogen peroxide [25]. Superoxide and hydrogen peroxide lead to the formation of secondary ROS, including hypochloric acid, singlet oxygen, hydroxyl radicals and peroxynitrite [25]. These ROS can directly damage the cell envelope, leading to lipid peroxidation as well as oxidative damage to WTA, LTA and the peptidoglycan [26,27]. In addition, ROS can damage the metabolic enzymes which *S. aureus* relies on for energy generation and macromolecule synthesis, for example, through the oxidation of the iron–sulphur clusters which are present in many metabolic enzymes [28]. Another innate immune component is anti-staphylococcal host fatty acids, such as long-chain unsaturated fatty acids. These activate the CtsR stress response, altering cellular metabolism, virulence factor production and surface properties, including leading to increased carotenoid biosynthesis and decreased surface hydrophobicity [29].

To survive inside the hostile environment, *S. aureus* encodes many regulatory systems for sensing and responding to host stresses [30]. The activation of many of these responses results in transcriptomic changes which then affect bacterial physiology. The stresses which can be sensed are diverse and include increased temperature, low pH, oxidative stress, host AMPs and nutrient limitation [31]. *S. aureus* responds to host AMPs and low pH by activating the GraRS two-component signalling system (TCS) [32,33]. This leads to the upregulation of *mprF* and the *dltABCD* operon, increasing positive surface charge [34]. Additionally, the high temperatures that result from pyrexia during infection lead to reduced rates of autolysis due to heat-induced damage to autolysins and their impaired targeting to the cell wall [35,36]. Physiological oxygen concentrations are also not replicated under standard laboratory conditions. At many sites of infection, for example, in deep skin and soft tissue infections or organ abscesses, oxygen is limited, exposing *S. aureus* to hypoxic conditions [37,38]. Physiological oxygen concentrations range between 1 and 11%, in contrast to the 20% typically used in laboratory experiments [37]. The low oxygen conditions are sensed by the SrrAB TCS, which induces expression of genes required for anaerobic growth [39,40]. As a facultative anaerobe, *S. aureus* can grow by anaerobic respiration or carbohydrate fermentation [41,42]. However, despite being able to grow in the absence of oxygen, the growth rate is much slower than in aerobic conditions [39].

## Host-induced cell envelope remodelling

The immune stress, nutritional composition and environmental conditions described above create a host environment that is fundamentally different from standard laboratory growth conditions [43,44]. One of the ways *S. aureus* responds to these stresses is by actively adapting its cell envelope, an extremely dynamic structure that is central to survival in the host. Many of these adaptations lead to aspects of the cell envelope being similar to those observed during the stationary phase. This section will focus on how the host environment induces remodelling of the staphylococcal cell envelope, while the next section will discuss the functional consequences of these changes on bacterial survival within the host.

## Cell membrane remodelling

**Fig. 1.**
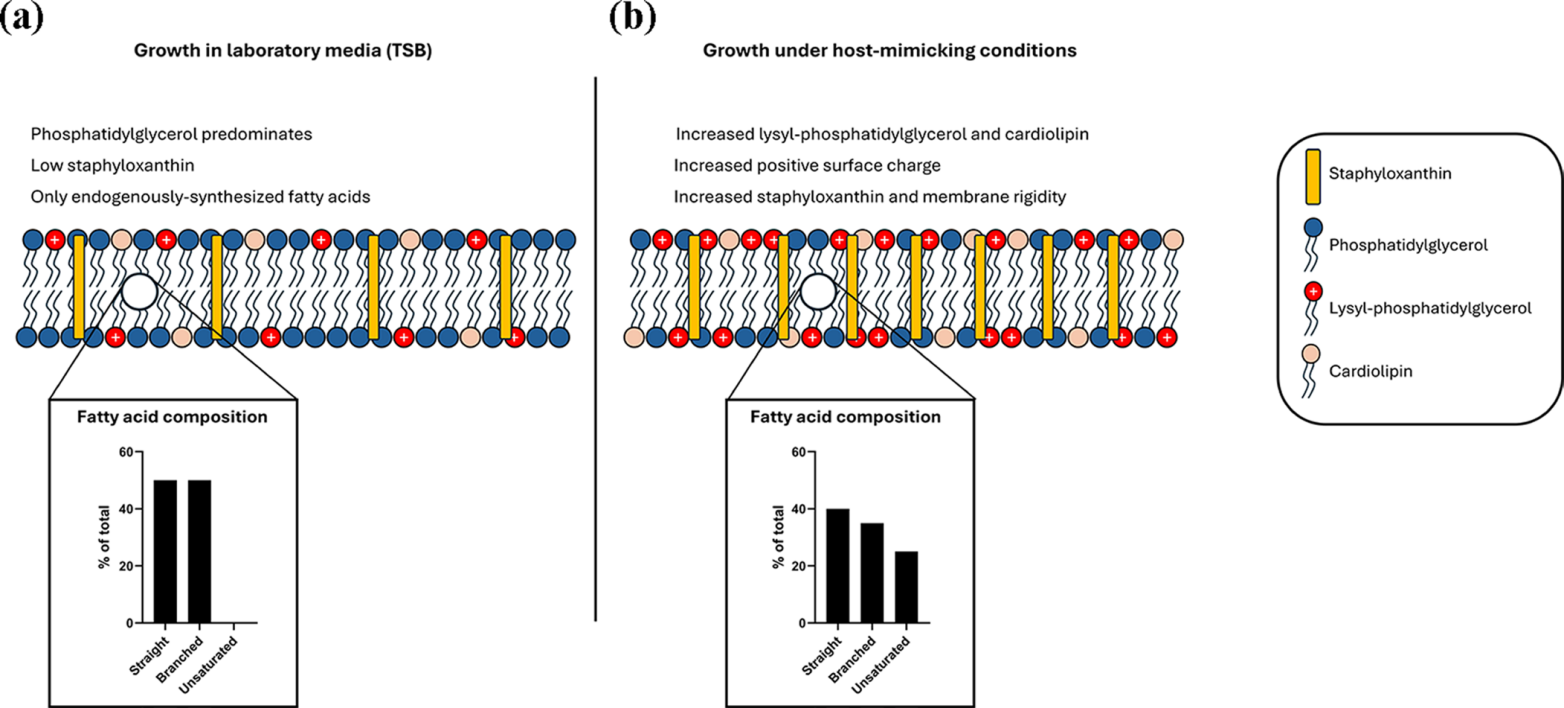
Host-induced changes in the staphylococcal cell membrane. The panel on the left (**a**) represents the cell membrane structure of exponential phase *S. aureus* when grown in tryptic soy broth (TSB), and the panel on the right (**b**) depicts the structure under host-mimicking conditions.

Three main factors that affect the properties of the staphylococcal membrane, the overall phospholipid composition, the fatty acid composition of the phospholipids and the staphyloxanthin content, are impacted by the host environment (Fig. 1) [9,45, 46].

### Lipid composition

When *S. aureus* is grown under laboratory conditions in tryptic soy broth (TSB), a standard growth medium, the most abundant phospholipid in the membrane is phosphatidylglycerol (PG) (~75% of total phospholipids) [45,47]. PG is the precursor for the two other main phospholipid species, lysyl-phosphatidylglycerol (LPG) and cardiolipin, which comprise ~15 and ~10% of phospholipids, respectively [47,48]. The phospholipid composition depends on the growth phase, with stationary phase cultures having increased cardiolipin and LPG and decreased PG compared to exponential phase cultures [45,49].

LPG is synthesized by MprF, which adds a lysyl group onto PG, conferring a positive charge on the phospholipid [50]. The expression of *mprF* is regulated by the GraRS TCS, which is activated by low pH and cationic AMPs including LL-37 and colistin [34]. In line with this, a mildly acidic pH of 5.5 led to a significant increase in LPG content in clinical *S. aureus* strains compared to at pH 7.4, with ~50% of total phospholipids being LPG [51]. Many epithelial sites within the host, along with the phagolysosomes of neutrophils and macrophages, are mildly acidic, meaning that it is plausible that the host environment may alter LPG content. Indeed, growth in human serum, which contains AMPs that activate GraRS, was found to lead to an increase in LPG content [52], and growth in mouse skin homogenate led to equal amounts of PG and LPG in contrast to more PG in TSB. Finally, both exposure to cell wall targeting antibiotics and growth in low oxygen conditions, as is found in many sites within the host, for example, in abscesses, have also been shown to increase LPG composition [53].

Cardiolipin is the third most abundant phospholipid and is synthesized from two molecules of PG by one of the two cardiolipin synthases, Cls1 and Cls2 [54,55]. These cardiolipin synthases are active under different conditions, with Cls2 being the main housekeeping cardiolipin synthase and playing a major role in the accumulation of cardiolipin during the stationary phase [55]. By contrast, Cls1 is responsible for cardiolipin synthesis under conditions of acid and salt stress [54,56]. In addition, other stresses including exposure to human serum, ATP deprivation and hypoxia also lead to increased cardiolipin content [54,57, 58]. However, the molecular mechanisms by which cardiolipin synthases are regulated are currently unknown.

### Fatty acid composition

*S. aureus* can synthesize fatty acids *de novo* using the fatty acid synthesis type II system. In the absence of exogenous fatty acids, phospholipids contain predominantly saturated fatty acids consisting of 14–20 carbon atoms, with 15–17 carbons the most common [59].

However, *S. aureus* can also take up fatty acids from the environment and incorporate them into its phospholipids using fatty acid kinase (FakAB) [60]. Exogenous fatty acids are bound by the fatty acid binding proteins FakB1 and FakB2 and then phosphorylated by FakA to create an acyl phosphate which is used for lipid synthesis [60]. Many niches within the host are rich in fatty acids, and these are preferentially used by *S. aureus* over endogenously synthesized fatty acids. *S. aureus* has been shown to use exogenous fatty acids from a range of host sources, including low-density lipoprotein (LDL), pig liver homogenate and mouse skin homogenate [61,62]. The use of these host-derived fatty acids results in *S. aureus* having very different fatty acid compositions under host conditions compared to in standard laboratory media [52,63, 64]. For example, while it is generally accepted that *S. aureus* cannot synthesize straight-chain unsaturated fatty acids (SCUFAs), cells grown in the presence of serum, liver extract and human LDL contain significant amounts of SCUFAs in their lipids [61,63]. Hines *et al*. found that these exogenous SCUFAs are incorporated into all classes of lipid, including PG, LPG, cardiolipin and glycolipids [52].

### Physicochemical properties of the membrane

Together, the lipid and fatty acid composition of the cytoplasmic membrane determine its physicochemical properties, including fluidity, surface charge and overall organization, all of which influence staphylococcal physiology and have consequences for survival in the host. A major factor impacting membrane fluidity is the fatty acid composition, with an increased ratio of branched-chain to straight-chain fatty acids reducing membrane rigidity, as branched fatty acids, synthesized from the branched-chain amino acids isoleucine, leucine and valine, pack less efficiently within the lipid bilayer [63,65,67]. As a result, membrane fluidity is highly sensitive to growth conditions and varies substantially between laboratory media and host-associated environments [63]. In particular, incorporation of host-derived SCUFAs, which are absent from membranes of *S. aureus* grown under standard laboratory conditions, can lead to an increase in membrane fluidity [63,68].

Membrane fluidity is also influenced by staphyloxanthin, the carotenoid pigment that gives *S. aureus* colonies their characteristic golden appearance, which intercalates into the membrane and decreases membrane fluidity due to its rigid diaponeurosporenoic group [69,70]. Due to its structure containing multiple conjugated double bonds, it facilitates the detoxification of ROS, protecting *S. aureus* from immune-mediated killing [70]. The regulation of staphyloxanthin production is complex, with factors affecting it including the alternative sigma factor SigB, the ssrA antisense RNA, metabolic status and the oxygen-responsive two-component system AirRS [71,75]. Staphyloxanthin levels are increased in the stationary phase and elevated under host-like conditions, with *S. aureus* grown in human serum exhibiting increased pigmentation compared to laboratory-grown cells [76,77]. In addition, steroid hormones such as dehydroepiandosterone have also been shown to increase carotenoid content and surface hydrophobicity [78]. However, non-pigmented variants frequently arise during infection, indicating that carotenoid production is dynamically regulated and may be dispensable in certain niches [79,80]. However, it is important to note that these variants that have been described are due to genetic mutations. It is currently unknown whether transient, non-genetic phenotypes occur in the host that are then lost on subsequent growth in laboratory media.

In addition to fluidity, membrane lipid composition influences surface charge, which plays a key role in interactions with cationic AMPs and antibiotics. Alterations in phospholipid composition, in particular increased LPG abundance, reduce the net negative charge of the membrane and contribute to resistance against host defence peptides [50]. The impact of altered surface charge is discussed in detail below.

Finally, the membrane is not uniform but is organized into structures known as functional membrane microdomains, regions of membrane that are rich in staphyloxanthin and scaffold proteins such as flotillin [81,83]. They play important roles in organizing protein complexes, including PBP2A and the Isd iron acquisition system and so affect antibiotic resistance and virulence [81,84]. However, how or whether the host environment impacts their formation, structure or number is currently unknown.

### Host lipid binding

One final major difference in the membrane of *S. aureus* grown in laboratory media compared to *in vivo* is the binding of host lipids, including phosphatidylcholine, phosphatidylethanolamine, sphingomyelins and cholesterol to the staphylococcal surface [52]. This lipid binding led to the extractable lipid content from *S. aureus* grown in serum being double that of when it was grown in media [52]. This was found to be due to lipids associating with the surface but not being incorporated into the membrane as they could be removed by washing with Triton X-100 and could be visualized as protrusions from the cell surface by electron microscopy [52]. Although the functional consequences of this lipid binding are currently unknown, these findings underscore the extent to which the envelope differs in the host from that formed under standard *in vitro* conditions.

## Cell wall remodelling

**Fig. 2.**
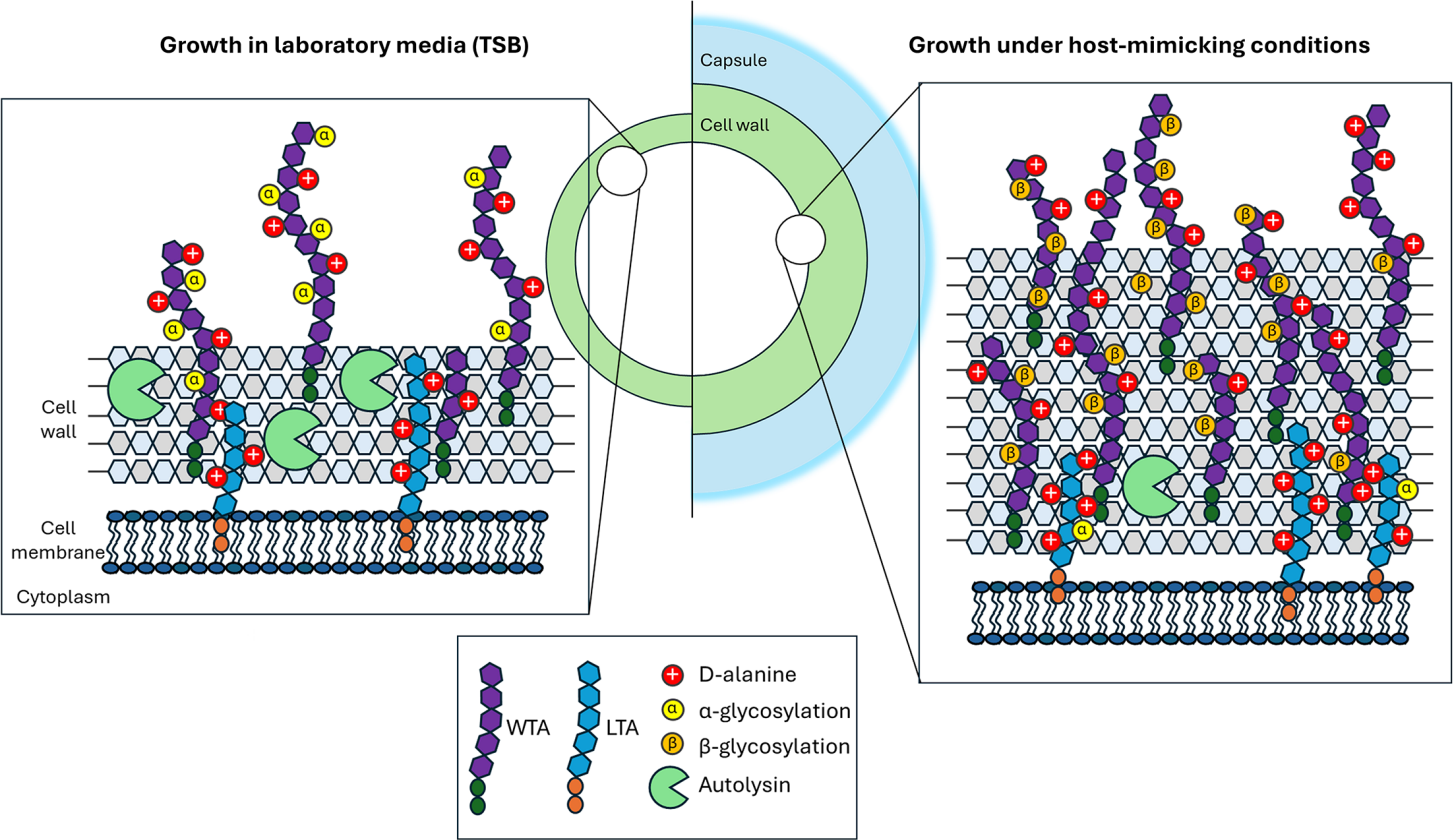
Host-induced changes in the staphylococcal cell wall. The panel on the left represents the cell wall structure of exponential phase *S. aureus* when grown in TSB and the panel on the right depicts the structure under host-mimicking conditions.

The structure of the cell wall when *S. aureus* is grown under standard laboratory conditions is fairly well characterized and has been the subject of recent reviews [4,5, 11], and this section discusses how host-induced cell wall remodelling affects each component of the cell wall (Fig. 2).

The staphylococcal cell wall is composed of the polymer peptidoglycan, which consists of glycan strands made of repeating disaccharide units of *N*-acetylglucosamine and *N*-acetylmuramic acid which are cross-linked by short peptides [4]. The average length of each glycan strand is six disaccharides, but this relatively short length is compensated for by the extremely high degree of crosslinking observed (typically 80–90%) [85,86]. The peptidoglycan is decorated with WTA, polysaccharides composed of repeating ribitol phosphate groups covalently bound to the muramic acid residues and proteins. It is estimated that in the stationary phase, peptidoglycan and WTA comprise ~80 and 20% of the mass of the cell wall, respectively [87]. However, this is affected by growth phase, with cells in the exponential phase having a higher WTA:peptidoglycan ratio [87]. WTAs are further modified by glycosylation and d-alanylation. In addition, many staphylococcal strains produce a polysaccharide capsule which is also covalently attached to the peptidoglycan.

### Peptidoglycan

One of the most dramatic aspects of host-induced cell wall remodelling is the increased cell wall thickness, a phenomenon which is also seen in the stationary phase cells [88]. This has been observed in *S. aureus* isolated from a murine kidney infection model, in *S. aureus* grown intracellularly inside endothelial cells and osteoblasts and in *S. aureus* grown in human serum [58,88, 89]. Studies using serum-grown cells found that this increased cell wall thickness was associated with increased levels of both peptidoglycan and WTA, with the extracted peptidoglycan from serum-grown cells having a fivefold higher dry weight than that from media-grown cells and containing fivefold higher levels of WTA [58].

Serum-induced cell wall thickening was found to be due to host AMPs, including the cathelicidin LL-37, activating the GraRS TCS system [58]. This positively regulates many genes involved in cell surface synthesis and modification, including *mprF,* which encodes the LPG synthase, and the *dltABCD* operon, which encodes the genes required for WTA d-alanylation [32,50, 90]. Cell wall thickening in the host is likely to be achieved by an altered balance between cell wall synthesis and cell wall degradation by peptidoglycan hydrolases [91]. Incubation in serum was found to lead to reduced hydrolase activity, as observed by zymography and Triton X-100-mediated autolysis assays [58]. Calprotectin, a protein produced by neutrophils, was also found to inhibit autolysins by sequestering the zinc they require for activity [92].

Peptidoglycan crosslinking is also affected by the host environment, although there are conflicting findings on how. Cells isolated from a murine kidney infection model were found to have reduced crosslinking compared to those grown in laboratory media, while no difference in crosslinking was observed between cells grown in human serum compared to media [58,88]. In addition, penicillin-binding protein 4, the protein that is responsible for the high degree of crosslinking in the staphylococcal cell wall, was found to be required for cell wall remodelling in serum, although whether it is its d-carboxypeptidase activity or its transpeptidase activity that was required is unknown [93]. These differences could be due to niche-specific differences within the host or host-specific differences between humans and mice. Alterations in peptidoglycan thickness and crosslinking are also likely to affect the mechanical properties of the cell wall, including stiffness and elasticity, which may influence susceptibility to immune-mediated damage and antibiotic penetration *in vivo*.

*S. aureus* peptidoglycan is modified by O-acetylation at the C-6 position of *N*-acetylmuramic acid, conferring protection against the host AMP lysozyme. The degree of O-acetylation varies depending on the strain (20–90%) but was not found to depend on the growth phase [94]. The expression of *oatA* is regulated by the GraRS TCS [34], and so this modification is likely to be affected by host stresses including low pH and AMPs, although the degree of peptidoglycan modification under host conditions is currently unknown.

Host-induced changes in nutrient availability and growth rate may also influence peptidoglycan turnover and recycling pathways, although whether PG recycling is actively remodelled during infection remains largely unexplored in *S. aureus*.

### Wall teichoic acids

Host-induced cell wall remodelling is associated with increased levels of WTA, polymers that play important roles in cell division, regulation of autolysins and bacterial adhesion [95]. This may be due to TcaA, as *tcaA* expression has been shown to be induced by serum and leads to increased ligation of WTA to the peptidoglycan [96]. WTAs are thought to protrude out from the peptidoglycan into the environment; however, how host-induced cell wall thickening affects WTA exposure is currently unknown [6]. Exposure to serum also leads to increased WTA d-alanylation, via GraRS-mediated upregulation of the *dltABCD* operon, reducing the net negative charge of the cell surface [58]. Finally, WTA glycosylation is heavily influenced by the environment; WTA can be glycosylated by two glycosyltransferases, TarM and TarS, which are responsible for the *α*- and *β*-GlcNAc substitutions, respectively [97,99]. However, it is important to note that while TarS is encoded by nearly all strains, many strains such as those from the emerging CC398 lack TarM [100]. While the *α*-GlcNAc modification dominates in *in vitro* grown *S. aureus*, this was found to almost completely switch to the *β*-GlcNAc modification in kidneys and livers of mice in a peritonitis model and in the muscles of mice infected in a deep wound model [101].

### Lipoteichoic acids

LTAs are membrane-bound polymers that are important for regulating cell morphology and division as well as impacting virulence and antibiotic resistance [7]. Compared to WTA, little is known about how they are affected by the host environment. Their length and abundance are tightly regulated and can be affected by the identity of the lipid anchor and the availability of starter units, with a PG anchor leading to longer polymers than a Glc_2_DAG anchor [102]. However, whether these factors impact LTA synthesis within the host environment is currently unknown. Modification of LTA, which occurs by both d-alanylation and glycosylation, is affected by host conditions [102]. The C2 hydroxyl of the LTA backbone can be modified either by the addition of a d-alanine or a GlcNAc group, and as these modifications occur at the same position, an increase in one modification can directly reduce the other [103]. Under laboratory conditions, LTA is highly modified with d-alanine, with ~70–85% of residues esterified [103,104]. d-Alanylation, mediated by the products of the *dlt* operon, is upregulated under conditions of cell envelope stress, including exposure to cationic AMPs and low pH [34,105]. By contrast, levels of glycosylation (mediated by YfhO) are very low under standard conditions (~2%) but increase under stress, including high salt or activation of SigB [103].

### Capsular polysaccharides

Many *S. aureus* strains produce a capsular polysaccharide, an important virulence factor that reduces complement deposition and neutrophil-mediated phagocytosis. Of the 13 capsular serotypes identified so far, only CP5- and CP8-expressing isolates have been associated with disease. Capsular polysaccharides are composed of repeating trisaccharide units containing *N*-acetylmannosaminuronic acid, *N*-acetyl-l-fucosamine and *N*-acetyl-d-fucosamine, and their biosynthesis is tightly regulated [106]. Capsule expression is controlled by a complex regulatory network comprising over 20 regulators involving global regulators such as CodY, SaeR and SigB [107,109]. Consistent with this complexity, capsule production is highly sensitive to environmental conditions, with substantial differences observed across growth phases and between standard laboratory growth and host-associated environments.

Several host-associated cues have been shown to modulate capsule expression. CO₂ concentration is a particularly important regulator, with growth under elevated CO₂ levels (1–5%), which reflect physiological concentrations in many host tissues, suppressing the expression of the *cap* operon and reducing the proportion of encapsulated cells [110,111]. In addition, capsule production is suppressed by both alkaline and anaerobic growth conditions [112]. *In vivo* studies support this, with analyses of isolates from cystic fibrosis patients and rat granuloma pouch models revealing that only a small fraction (∼1–5%) of bacteria expressed capsule *in situ*, whereas the same isolates reverted to high levels of capsule expression (70–90%) when cultured aerobically *in vitro* [111]. Iron limitation, another hallmark of the host environment, has also been shown to increase capsule production [113], linking nutritional immunity directly to surface architecture. Importantly, capsule expression is not stable, with multiple studies demonstrating that strains appearing acapsular *in vitro* can regain capsule expression following passage through animal infection models, including mouse bacteraemia, although the mechanisms driving this phenotypic switching remain poorly understood [114,116].

### Host protein binding

In addition to intrinsic remodelling of the cell envelope, *S. aureus* can further modify its surface through the binding of host proteins, concealing the underlying cell envelope. A wide range of host factors, including fibrinogen, fibronectin, vitronectin, immunoglobulins and complement components, can associate with the staphylococcal surface, either through specific interactions with surface-anchored proteins or via non-specific adsorption [117,121]. This host-derived protein layer can mask immunogenic envelope components, reduce opsonophagocytic killing and promote immune evasion [122]. Although host protein binding is primarily mediated by surface proteins rather than changes in envelope biosynthesis, it is likely to act synergistically with capsule production, altered teichoic acid composition and cell wall thickening to affect the physiology and functional properties of the staphylococcal surface during infection. A detailed discussion of staphylococcal adhesins and host protein interactions is beyond the scope of this review but has been comprehensively covered elsewhere [123,124].

## Consequences of cell envelope remodelling

**Fig. 3.**
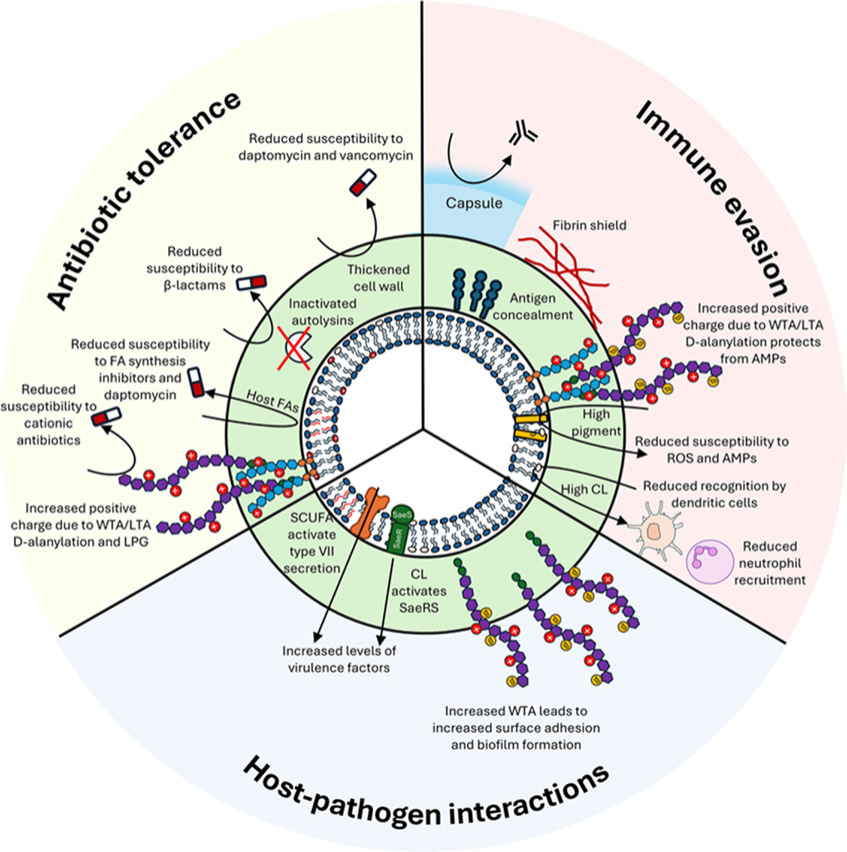
Functional consequences of host-induced cell envelope remodelling.

The cell envelope is the main target for many antibiotics, contains the targets recognized by the immune system and mediates interactions with host cells and tissues that enable dissemination and disease. Host-induced changes to the bacterial cell surface therefore have profound implications for antibiotic susceptibility, susceptibility to the immune system and staphylococcal pathogenesis (Fig. 3).

### Altered susceptibility to antibiotics

Many of our most successful and commonly used antibiotics target the staphylococcal cell envelope [125,126]. Beta-lactams and vancomycin inhibit the transpeptidation step of peptidoglycan synthesis by binding the penicillin-binding proteins and the d-ala-d-ala motif of lipid II, respectively, while daptomycin damages the cell membrane and inhibits cell wall synthesis, possibly by disrupting the localization of the cell division machinery [125,126].

One of the best characterized examples of how cell envelope remodelling affects antibiotic susceptibility is the impact of increased cell wall thickness and altered crosslinking on vancomycin susceptibility. Such isolates, known as vancomycin-intermediate *S. aureus* (VISA), show reduced susceptibility to vancomycin due to their thickened cell wall trapping vancomycin within the cell wall and preventing it from reaching lipid II [127,128]. Similarly, reduced crosslinking increases the number of free d-ala-d-ala motifs present in the wall, which act as decoys and further bind vancomycin. However, the phenotypes of these VISA isolates are due to genetic mutations, frequently in *walKR*, *graRS, vraSR* and *rpoB*, rather than being induced by the environment [129,131]. By contrast, host-induced changes typically confer phenotypic antibiotic tolerance rather than stable genetic resistance, reducing killing without altering MIC values measured under standard laboratory conditions [132]. However, the thickened cell wall of VISA isolates is reminiscent of the thickened cell wall which has been found to be triggered by the host environment [52,58, 88]. In line with this, human serum-induced cell wall thickening was found to protect *S. aureus* from vancomycin [58].

However, this cell wall thickening not only protects from vancomycin but also other antibiotics from a range of classes and with various mechanisms of action, including daptomycin, gentamicin and oxacillin [14,58]. This increased daptomycin tolerance was found to be due to the thickened cell wall impeding penetration of daptomycin and so reducing its ability to reach and bind to its membrane target [14,58]. In addition, the thickened cell wall was found to be associated with an increased WTA content, which, due to its modification with positively charged d-alanine residues, reduces the net negative charge of the cell surface [58]. This altered charge was proposed to have been the mechanism of the reduced gentamicin susceptibility, as this antibiotic is cationic [58]. In parallel, the increased d-alanylation of LTA under conditions of cell envelope stress also reduces the net negative charge of the cell surface, possibly contributing to decreased susceptibility to cationic antimicrobials. Recently, host-imposed nutritional immunity, specifically the sequestration of zinc ions by calprotectin, was found to inactivate Atl, the main peptidoglycan hydrolase in *S. aureus*, leading to reduced susceptibility to *β*-lactams including oxacillin [92].

Changes to the cell membrane also alter antibiotic susceptibility. Here, the most well-studied example is the activation of the GraRS stress response by low pH and the presence of AMPs, leading to increased MprF, the enzyme responsible for the synthesis of LPG and flipping it to the outer leaflet of the membrane [105,133]. Again, this reduces the net negative charge of the membrane, reducing susceptibility to cationic antibiotics including daptomycin [126,133]. Serum-induced changes in membrane composition, specifically an increased cardiolipin content, were also found to contribute to daptomycin susceptibility [58].

Membrane fluidity is also proposed to affect antibiotic susceptibility, especially daptomycin susceptibility [69,134, 135]. The mechanisms behind this are not fully clear, although it may be due to altered fluidity affecting the ability of daptomycin to form pores and permeabilize the membrane [134]. Moreover, there is no consensus on whether it is increased or decreased fluidity that mediates reduced susceptibility, with an analysis of ten clinical bloodstream susceptible/resistant strain pairs, demonstrating increased fluidity in resistant strains [69], while a 20-day serial daptomycin exposure experiment resulted in non-susceptible strains with decreased membrane fluidity due to an increase in staphyloxanthin content [136]. These opposing findings may partly reflect differences between *in vivo* adaptation and *in vitro* selection, which impose distinct evolutionary pressures and so lead to different resistance mechanisms. Importantly, these studies describe genetically adapted strains, whereas host-induced membrane remodelling is phenotypic and reversible. Under host-mimicking conditions, incorporation of host-derived unsaturated fatty acids increases membrane fluidity, while increases in staphyloxanthin reduce membrane fluidity [63]. As such, predicting the overall impact of host-induced membrane remodelling on daptomycin susceptibility remains challenging.

Finally, the incorporation of host fatty acids into staphylococcal phospholipids has been shown to bypass the requirements for endogenous fatty acid synthesis, promoting resistance to antimicrobials which target fatty acid synthesis, such as the biocide triclosan [137,138]. By contrast, the incorporation of polyunsaturated fatty acids, for example, the host-derived fatty acid arachidonic acid, leads to an increased susceptibility to aminoglycoside antibiotics by facilitating the antibiotic crossing the membrane and entering the cell [139].

### Altered susceptibility to immune killing

Host-induced changes in the cell envelope also have significant implications for the susceptibility of *S. aureus* to various components of the immune system. The most important cell type for controlling *S. aureus* infections is neutrophils, which phagocytose antibody- and complement-opsonized bacteria and kill them via mechanisms including the production of AMPs and ROS [10]. To be efficiently phagocytosed, bacteria must be opsonized with antibody and/or complement. The mechanisms used by *S. aureus* to evade this are numerous and have been well characterized [140]; they include production of the Fc-binding proteins Protein A and Sbi and interfering with the complement cascade using complement factor I and factor H and SCIN [141]. However, in addition to these mechanisms that are genetically encoded, there is growing evidence that host-induced remodelling of the cell envelope provides an additional, dynamic layer of immune evasion, affecting both phagocytosis and survival inside the neutrophil.

One way that *S. aureus* protects itself from phagocytosis is by coating itself with host proteins. For example, extracellular fibrinogen-binding protein (Efb) binds host fibrinogen, generating a shield-like structure that prevents phagocytic receptors from recognizing surface-bound C3b and antibodies [122]. Similarly, *S. aureus* can cleave fibrinogen to form fibrin using two coagulases, coagulase (Coa) and von Willebrand factor-binding protein (vWbp), encasing *S. aureus* in a fibrin shield which protects the bacterium from clearance by neutrophils [142].

More recently, it has been found that GraRS-mediated cell wall thickening conceals many of the surface proteins, including membrane proteins and cell wall-associated proteins, reducing the ability of antibody to bind and opsonize the staphylococcal surface, protecting the bacteria from neutrophil-mediated phagocytosis [143]. WTA can play a similar role, preventing antibodies from recognizing and opsonizing the cell wall and reducing the recognition of peptidoglycan by toll-like receptors [144]. However, WTAs are also immunogenic and can be presented to T cells [144], and so how the increased WTA observed under host conditions affects susceptibility to the immune response remains to be determined. WTA glycosylation further impacts recognition by the immune system, as, likely reflecting the increased relative abundance of *β*-glycosylated WTA compared to *α*-glycosylated under host conditions, human serum contains a tenfold higher concentration of antibodies recognizing *β*-glycosylated WTA than the *α*-glycosylated form [145].

Many host AMPs, including the human cathelicidin LL-37 and the *α*- and *β*-defensins, are cationic [146]. This means that, similarly to the cationic antibiotic daptomycin, susceptibility to these peptides is dramatically affected by the surface charge of the bacteria. As described above, the stresses within the host environment lead to increased levels of membrane LPG and an increase in the amount of d-alanylated teichoic acids, increasing the surface positive charge and protecting *S. aureus* from cationic AMPs [50,52, 58]. WTA d-alanylation is crucial for mediating susceptibility to neutrophil-produced defensins, with a mutant lacking this modification efficiently killed by neutrophils even in the absence of the oxidative burst [147]. The susceptibility to AMPs is also affected by the membrane fluidity, with more rigid membranes protecting *S. aureus* from AMPs including magainin and gramicidin [148]. As well as increasing membrane rigidity, the increased level of staphyloxanthin found in *S. aureus* grown under host conditions may also contribute to intracellular survival as it is a potent antioxidant, protecting the bacteria from the activity of ROS [149,150].

As well as being less susceptible to membrane-targeting antimicrobials, *S. aureus* strains with increased levels of cardiolipin in their membranes have been shown to elicit weaker immune responses [151,152]. These strains led to the recruitment of fewer neutrophils to the site of infection [152] and were less well recognized by dendritic cells, resulting in a reduced induction of cytokine responses [151].

However, not all host-environment-associated changes in the cell surface contribute to immune evasion; for example, the incorporation of host SCUFAs has been shown to lead to increased Toll-like receptor 2-mediated recognition of *S. aureus* by the innate immune system [153].

### Altered host–pathogen interactions

Finally, host-induced cell wall remodelling is likely to have a significant impact on the way that *S. aureus* interacts with the host more broadly beyond the immune system; however, this has not yet been well characterized or studied.

One major area where this is seen is in bacterial adhesion to host cells. For example, the increased levels of WTA present after host-induced cell wall remodelling are likely to impact the binding of *S. aureus* to surfaces and biofilm formation. WTAs are crucial for the adherence of *S. aureus* to artificial surfaces including glass and polystyrene, and mutants lacking WTA are impaired in biofilm formation [154,157]. It has recently been shown that *S. aureus* uses serum triglycerides to support increased WTA synthesis, promoting increased biofilm formation and tolerance to vancomycin and daptomycin [158]. While glycosylation was not found to be important for this process, TarM-catalysed *α*-glycosylation has been shown to be important for the binding of *S. aureus* to epithelial cells during colonization [159].

Secondly, changes in the cell surface, particularly host-induced alterations in surface protein exposure and in WTA abundance, are likely to impact the interaction of *S. aureus* with host cells. For example, WTAs interact with various host receptors, including the C-type lectin receptors langerin and macrophage galactose-type lectin, affecting the activation of antigen-presenting cells [6]. However, how the host-mediated increase in WTA affects the interaction of *S. aureus* with antigen-presenting cells is currently unknown.

Finally, host-induced cell envelope remodelling impacts bacterial virulence. The decreased membrane fluidity resulting from the incorporation of host SCUFAs into *S. aureus* phospholipids was found to activate the type VII secretion system, a system which impacts virulence and is required for abscess formation in murine models [160]. Moreover, cardiolipin regulates the expression of eight TCSs in *S. aureus*, including SaeRS, a system which regulates key virulence factors including haemolysins and coagulases, meaning that the increased cardiolipin levels found under host conditions could impact bacterial virulence via these pathways [161].

## Conclusions and future perspectives

As outlined in this review, the staphylococcal cell envelope is profoundly influenced by the host environment during infection, with exposure to host-imposed stresses driving extensive remodelling of both the membrane and the cell wall. Fully characterizing these changes and their impact is crucial as they alter susceptibility to antibiotics and the immune system as well as affecting pathogenicity. However, as this cell envelope remodelling is phenotypic rather than resulting from genetic changes, its consequences are unlikely to be detected by routine diagnostics yet may contribute to treatment failure and the development of chronic or relapsing infection.

Many aspects of cell wall remodelling can be inhibited by cell wall synthesis inhibitors such as *β*-lactams or fosfomycin, raising the possibility that a combination approach of these drugs with other antibiotics such as vancomycin or daptomycin may be useful. At the moment, there is a lack of large-scale evidence as to whether combination therapy is more effective; however, this is currently being tested by the SNAP trial, which aims to determine whether cefazolin improves treatment outcomes in patients treated with vancomycin or daptomycin [162]. However, several small-scale trials support the combination approach with patients treated with dual therapy suffering from lower rates of treatment failure than monotherapy [163,165]. In addition to improving the efficacy of other antibiotics, inhibiting cell wall remodelling may improve the ability of the immune system to clear infection by enhancing opsonization and increasing neutrophil-mediated killing.

It is important to note that different niches within the host impose different combinations of stresses on the bacterium. For example, the bloodstream exposes bacteria to high concentrations of AMPs, intracellular niches impose acidic and oxidative stresses and deep tissue abscesses are often hypoxic. Therefore, the precise architecture of the cell surface is likely to vary substantially between different infection sites, possibly contributing to differences in treatment outcomes between infection types. It also raises the possibility that different therapeutic approaches and combinations may be optimal for different infections, as antibiotics that are effective against bacteria in one physiological state may be less effective in another. A better understanding of how each specific host niche affects the staphylococcal cell envelope will therefore be essential for developing better therapeutic approaches.

Studying host-induced cell wall remodelling and its consequences is challenging as isolating sufficient bacteria to study from infected patients can be difficult and phenotypes which were induced in the host are subsequently lost on culturing the bacteria in laboratory media. Animal models may help; however, they do not fully recapitulate human infections, with important differences existing between immune responses and the host stresses imposed [166]. For example, the diversity and activity of AMPs vary significantly between species [167]. Moreover, many *in vivo* infection models rely on high inocula and infection routes that do not accurately reflect the establishment and progression of natural human infection [166]. Addressing this will require the development of improved experimental approaches and host-mimicking *in vitro* systems or methods to analyse the cell surface architecture of bacteria isolated directly from clinical samples.

**Box 1. Outstanding questions.**

1. What is the cell envelope architecture *in vivo*?
2. How does this cell envelope architecture vary across infection niches?
3. What are the extent and dynamics of remodelling during infection and how is this coordinated in response to multiple host cues?
4. Which cell envelope changes have the largest impact on infection outcomes and can these be targeted therapeutically?

Despite increasing recognition that the host environment profoundly remodels the *S. aureus* cell envelope, many fundamental questions remain unanswered. For example, the regulatory mechanisms that sense host-derived cues and coordinate envelope remodelling are still poorly understood. Moreover, how the multiple host stresses are integrated at the regulatory level to produce specific envelope states remains unclear. Furthermore, due to the challenges described above, most current knowledge derives from simplified *in vitro* systems that only approximate aspects of the host environment, meaning that the extent, dynamics and heterogeneity of envelope remodelling during infection remain largely unknown. It is also unclear how rapidly these changes occur, whether distinct bacterial subpopulations adopt different envelope states within the same infection niche and how stable these phenotypes are over the course of infection. In addition, while several envelope modifications have been linked to altered antibiotic tolerance or immune evasion, the relative contribution of each change to infection outcomes has not yet been defined. Together, recognizing the importance and consequences of host-driven envelope remodelling will be critical for gaining a deeper understanding of staphylococcal pathogenicity and to designing better treatment strategies to improve patient outcomes.

## Abbreviations

AMP: antimicrobial peptide
LDL: low-density lipoprotein
LPG: lysyl-phosphatidylglycerol
LTA: lipoteichoic acid
PG: phosphatidylglycerol
ROS: reactive oxygen species
SCUFA: straight-chain unsaturated fatty acid
TCS: two-component signalling system
TSB: tryptic soy broth
VISA: vancomycin-intermediate *Staphylococcus aureus*
WTA: wall teichoic acid
